# Supplementary material for: Heat adaptation of phage T7 under an extended genetic code
Source: Virus Evol. 2021 Dec 1;7(2):veab100. doi: 10.1093/ve/veab100 (PMC8923235; doi:10.1093/ve/veab100)
Supplement: veab100_Supp [file veab100_supp.zip › Sequencing_Analysis.zip!/Sequencing_Analysis/A1/evidence/SNP_9.html]

BRESEQ :: Evidence


| Predicted mutation | | | | | | |
| --- | --- | --- | --- | --- | --- | --- |
| evidence | seq id | position | mutation | annotation | gene | description |
| RA | NC\_001604.1 | 28,691 | A→C | intergenic (–/–) | – / – | –/– |

| Read alignment evidence... | | | | | | | | | | | |
| --- | --- | --- | --- | --- | --- | --- | --- | --- | --- | --- | --- |
|  | seq id | position | | ref | new | freq | score (cons/poly) | reads | annotation | genes | product |
| \* | NC\_001604.1 | 28,691 | 0 | A | C | 98.1% | 8891.6 / NA | 2922 | intergenic (–/–) | *–/–* | –/– |
| Reads supporting (aligned to +/- strand):  **ref** base A (22/33);  **new** base C (1369/1479);  **total** (1397/1525) | | | | | | | | | | | |
| Fisher's exact test for biased strand distribution *p*-value = 2.76e-01 | | | | | | | | | | | |
| Kolmogorov-Smirnov test that lower quality scores support variant *p*-value = 1.00e+00 | | | | | | | | | | | |

Only 100 of 2974 total aligned reads displayed.  

`GGAGGTATGGGCTATCGTGCAGCAACCACTCAGGCCGAACAGCCAAGGTCAAGCCTATTGGACACCATTGGTCGGTTCGCTAAGGCTGGTGCCGATATGTATACCGCTAAGGAACAACGAGCACGAGACCTAGCTGATGAACGCTCTAACGAGATTATCCGTAAGCTGACCCCTGAGCAACGTCGAGAAGCTCTCAACAACGGGACCCTTCTGTATCAGGATGACCCATACGCTATGGAAGCACTCCGAGTCAAGACTGGTCGTAACGCTGCGTATCTTGTGGACGATGACGTTATGCAGAAGATAAAAGAGGGTGTCTTCCGTACTCGCGAAGAGATGGAAGAGTATCGCCATAGTCGCCTTCAAGAGGGCGCTAAGGTATACGCTGAGCAGTTCGGCATCGACCCTGAGGACGTTGATTATCAGCGTGGTTTCAACGGGGACATTACCGAGCGTAACATCTCGCTGTATGGTGCGCATGATAACTTCTTGAGCCAGCAAGCTCAGAAGGGCGCTATCATGAACAGCCGAGTGGAACTCAACGGTGTCCTTCAAGACCCTGATATGCTGCGTC‑GTCCAGACTCTGCTGACTTCTTTGAGA  >  NC_001604.1/28391‑28991`  

                                                                                                                                                                                                                                                                                                            `|`                                                                                                                                                                                                                                                                                                               
         `ggCTATCGTGCAGCAACCACGCAGGCCGAACAGCCAATGTCAAGCCTATTGGACACCATTGGTCGGTTCGCTAAGGCTGGTGCCGATATGTATACCGCTAAGGAACAACGAGCACGAGACCTAGCGGATGAACGCTCTAACGAGATTATCCGTAAGCTGACCCCTGAGCAACGTCGAGAAGCTCTCAACAACGGGACCCTTCTGTATCAGGAAGACCCATACGCTATGGAAGCACTCCGAGTCAAGACTGGTCGTAACGCTGCGTATCTTGTGGACGATGACGTTATGCAGCAGATAAAag                                                                                                                                                                                                                                                                                                      <  1:469522/301‑1 (MQ=255)`  
         `ggCTAGCGGGCAGCAAACAATAAGGACGAACAGCGAAGGTCAAGCGTATTGGACACCATTGGGCGGTTAGCGAAGGCTGGTGACAATAGGTAGAACGATAAGGAAGAACGAGAACGAGACCTAGCTGATGAACGCTCTAACGAGATTAACCGTAAGCTGAACAATGAGCAACGTCGAGAAGCTCTCAACAACGGGAACCTTCTGTATCAGGAGGACCCAGAAGCTATGGAAGCACACCGAGTAAAGACTGGTCGTAACGCTGCGTATCTTGGGGACGATGACGTTATGCAGAAGATAAAag                                                                                                                                                                                                                                                                                                      <  1:71081/301‑1 (MQ=255)`  
          `gCTATCGTGCAGCAACCACTCAGGCCGAACAGCCAAGGTCAAGCCTATTGGACACCATTGGTCGGTTCGCTAAGGCTGGTGCCGATATGTATACCGCTAAGGAACAACGAGCACGAGACCTAGCTGATGAACGCTCTAACGAGATTATCCGTAAGCTGACCCCTGAGCAACGTCGAGAAGCTCTCAACAACGGGACCCTTCTGTATCAGGATGACCCATACGCTATGGAAGCACTCCGAGTCAAGACTGGTCGTAACGCTGCGTATCTTGTGGACGATGACGTTATGCAGCAGATAAAaga                                                                                                                                                                                                                                                                                                     <  1:50346/301‑1 (MQ=255)`  
           `ctagctggcagcaACCACTAAGGCCGAACAGCCAAGGTCAAGCCTATTGGACACCATTGGTCGGTTCGCTAAGGCTGGTGCCGATATGTATGCCGCTAAGGAACAACGAGCACGAGACCTAGCTGATGAACGCTCTAACTAGATTATCCGTAAGCTGACCCCTGAGCAACGTCGAGAAGCTCTCAACAACGGGACCCTTCTGTATCAGGATGACCCATACGCTATGGAAGCACTCCGAGTCAAGACTGGTCGTAACGCTGCGTATCTTGTGGACGATGACGTTATACAGCAGATAAAagag                                                                                                                                                                                                                                                                                                    <  1:506753/294‑1 (MQ=255)`  
             `aTCGTGCAGCAACCACTCAGGCCGATCAGCCAAGGTCAAGCCTATTGGACACCATTGGTCGGTTCGCTAAGGCTGGTGCCGATATGTATGCCGCTAAGGAACAACGAGCACGAGACCTATCTGATGAACGCTCTAACGAGATTATCCGTAAGCTGACCCCTGAGCAACGTCGAGAAGCTCCCACCAACGGGACCCTTCTGTCTCTGGATGACCCATACGCTATGGAAGCACTCCGAGTCAAGACTGGTCTTAACGCTGCGTATCTTGTGGCCGATGACGTTATGCATCAGATAAAAGAtgg                                                                                                                                                                                                                                                                                                  >  1:397422/1‑301 (MQ=255)`  
             `aTCGTGCAGCAACCACTCAGGCCGAACAGCCAAGGTCAAGCCTATTGGACACCATTTGTCGGTTCGCTAAGGCTGGTGCCGATATGTATGCCGCTAAGGAACAACGAGCACGAGACCTAGCTGATGAACGCTCTAACAAGATTATCCGTAAGCTGACCCCTGAGCAACGTCGAGAAGCTCTCAACAACGGGACCCTTCTGTATCAGGATGACCCATACGCTATGGCAGCACCCCGAGTCAAGACTGGTCTTAACGCTGCGTATCTTGTGGACGATGACGTTATGCAGCAGATAAAaggtgt                                                                                                                                                                                                                                                                                                  >  1:228050/1‑297 (MQ=255)`  
             `aTCGTGCAGCAACCACTCAGGCCGAACAGCCAAGGTCAAGCCTATTGGACACCATTGGTCGGTTCGCTAAGGCTGGTGCCGATATGTATGCCGCTAAGGAACAACGAGCACGAGACCTAGCTGATGAACGCTCTAACGAGATTATCCGTAAGCTGACCCCTGAGCAACGTCGAGAAGCTCTCAACAACGGGACCCTTCTGTATCAGGATGACCCATACGCTATGGAAGCACTCCGAGTCAAGACTGGTCGTAACGCTGCGTATCTTGTGGACGATGACGTTATGCAGCAGATaaactgtct                                                                                                                                                                                                                                                                                                  >  1:377211/1‑295 (MQ=255)`  
                    `agcaACCACTCAGGCCGAACAGCCAAGGTCAAGCCTATTGGACACCATTGGTCGGTTCGCTAAGGCTGGTGCCGATATGTATGCCGCTAAGGAACAACGAGCACGAGACCTAGCTGATGAACGCTCTAACGAGATTATCCGTAAGCTGACCCCTGAGCAACGTCGAGAAGCTCTCAACAACGGGACCCTTCTGTATCAGGATGACCCATACGCTATGGAAGCACTCCGAGTCAAGACTGGTCGTAACGCTGCGTATCTTGTGGACGATGACGTTATGCAGCAGATAAAAGAGGGTGTCTTc                                                                                                                                                                                                                                                                                           <  1:437060/301‑1 (MQ=255)`  
                       `cagcACTTAGGCCGAACAGCCAAGGTCAAGCCTATTGGACACCATTGGTCGGGTCGCTAAGGCTGGTGCAGATATGTATGACGCTAAGGAACAACGAGCACGAGACCTAGCTGATGAACGCTCTAACGAGATTATCCGTAAGCTGACCCCTGAGCAACGTCGAGAAGCTCTCAACAACGGGACCCTTCTGTATCAGGATGACCCATACGCTATGGAAGCACTCCGAGTCAAGACTGGTCGTAACGCTGCGTATCTTGTGGACGATGACGTTATGCAGCAGATAAAAGAGGGTGTCTTCCGt                                                                                                                                                                                                                                                                                        <  1:186232/298‑1 (MQ=255)`  
                          `cACTCAGGCCGAACAGCCAAGGTCAAGCCTATTGGACACCATTGGTCGGTTCGCTAAGGCTGGTGTCGATATGTATACCGCTAAGGAACAACGAGCACGAGACCTAGCTGATGAACGCTCTAACGAGATTATCCGTAAGCTGACCCCTGAGCAACGTCGAGAAGCTCTCAACAACGGGACCCTTCTGTATCAGGATGACCCATACGCTATGGAAGCACTCCGAGTCAAGACTGGTCGTAACGCTGCGTATCTTGTGGACGATGACGTTATGCAGCAGATAAAAGAGGGTGTCTTCCGTACt                                                                                                                                                                                                                                                                                     <  1:75332/301‑1 (MQ=255)`  
                           `aCTCAGGCCGAACAGCCAAGGTCAAGCCTATTGGACACCATTGGTCGGTTCGCTAAGGCTGGTGCCGATATGTATGCCGCTAAGGAACAACGAGCACGAGACCTAGCTGATGAACGCTCTAACGAGATTATCCGTAAGCTGACCCCTGAGCAACGTCGAGAAGCTCTCAACAACGGGACCCTTCTGTATCAGGATGACCCATACGCCATGGAAGCACTCCGAGTCAAGACTGGTCGTAACGCTGCGTATCTTGTGGACGATGACGTTATGCAGCAGATAAAAGAGGgtgctgccccttata                                                                                                                                                                                                                                                                                    >  1:1646/1‑289 (MQ=255)`  
                                `ggCCGAACAGCCAAGGTCAAGCCTATTGGACACCATTGGTCGGTTCGCTAAGGCTGGTGCCGATATGTATGCCGCTAAGGAACAACGAGCACGAGACCTAGCTGATGAACGCTCTAACGAGATTATCCGTAAGCTGACCCCTGAGCAACGTCGAGAAGCTCTCAACAACGGGACCCTTCTGTATCAGGATGACCCATACGCTATGGAAGCACTCCGAGTCAAGACTGGTCGTAACGCTGCGTATCTTGTGGACGATGACGTTATGCAGCAGATAAAagagctgtctcttatacacatctcc                                                                                                                                                                                                                                                                               >  1:307826/1‑280 (MQ=255)`  
                                          `ccAAGGTCAAGCCTATTGGACACCATTGGTCGGGTCGCTAAGGCTGGTGCCGATATGTATACCGCTAAGGAACAACGAGCACGAGACCTAGCTGATGAACGCTCTAACGAGATTATCCGTAATCTGACCCCTGAGCAACGTCTAGAAGCTCTCAACAACGGGACCCTTCTGTATCAGGATGACCCATACGCTATGGAAGCACTCCGAGTCAAGACTGGTCGTAACGCTGCGTATCTTGTGGACGATGACGTTATGCAGCAGATAAAAGAGGGTGTCTTCCGTACTCGCGAAGAGATGGAag                                                                                                                                                                                                                                                                     <  1:288912/301‑1 (MQ=255)`  
                                          `ccAAGGTCAAGCATATTGGACACCATTGGTCGGTTCGCTAAGGCTGGTGCCGATATGTATACCGATAAGGAACAACGAGCACGAGACCTAGCTGATGAACGCTCTAACGAGATTATCCGTAAGCTGACCCCTGAGCAACGTCGAGAAGCTCTCAACAACGGGACCCTTCTGTATCAGGATGACCCATACGCTATGGAAGCACTCCGAGTCAAGACTGGTCGTAACGCTGCGTATCTTGTGGACGATGACGTTATGCAGCAGATAAAAGAGGGTGTCTTCCGTACTCGCGAAGAGATGGAag                                                                                                                                                                                                                                                                     <  1:486270/301‑1 (MQ=255)`  
                                               `gTCAAGCCTATTTTACACCATTGGTCGGTTCGCTAAGGCTGGTGCCGATATGTATACCGCTAAGGAACAACGAGCACGAGACCTAGCTGATGAACGCTCTAACGAGATTATCCGTAAGCTGACCCCTGAGCAACGTCGAGAAGCTCTCAACAACGGGACCCTTCTGTATCAGGATGACCCATACGCTATGGAAGCACTCCGAGTCAAGACTGGTCGTAACGCTGCGTATCTTGTGGACGATGACGTTATGCAGCAGATAAAAGAGGGTGTCTTCCGTACTCGCGAAGAGATGGAAGAGTAt                                                                                                                                                                                                                                                                <  1:100210/301‑1 (MQ=255)`  
                                               `atgtgtataagagacagccATTGGTCGGTTCGCTAAGGCTGGTGCCGATATGTATGCCGCTAAGGAACAACGAGCACGAGACCTAGCTGATGAACGCTCTAACGAGATTATCCGTAAGCTGACCCCTGAGCAACGTCGAGAAGCTCTCAACAACGGGACCCTTCTGTATCAGGATGACCCATACGCTATGGAAGCACTCCGAGTCAAGACTGGTCGTAACGCTGCGTATCTTGTGGACGATGACGTTATGCAGCAGATAAAAGAGGGTGTCTTCCGTACTCGCGAAGAGATGGAAGAGTAt                                                                                                                                                                                                                                                                <  1:373317/284‑1 (MQ=255)`  
                                                   `aGCCTATTGGACACCATTGGTCGGTTCGCTAAGGCTGGTGCCGCGATGTATACCGCTAAGGAAAAACGAGCATGAGACCTAGCTGATGAACGCTCTAACGAGATTAACCGTAAGCTGACCCCTGAGCAACGTCGAGAAGCCCTCAACAACGGGACCCTTCTGTAGCAGGATGACCAATACGCTATGGAAGAACTAAGAGTTAAGACTGGACGTAAAGCAGCGTATCTTGCGGAAGATGACGCTATGCAGCAGATAATAGAGGGTGTCTACCGTACTcggtagtagatgaaagcatgttctt                                                                                                                                                                                                                                                            >  1:256820/1‑278 (MQ=255)`  
                                                   `aGCCTATTGGACACCATTGGTCGGTTCGCTAAGGCTGGTGCCGATATGTATGCCGCTAAGGAACAACGAGCACGAGACCTAGCTGATGAACGCTCTAACGAGATTATCCGTAAGCTGACCCCTGAGCAACGTCTAGAAGCTCTCAACAACGGGACCCTTCTGTATCAGGATGACCCATACGCTATGGAAGCACTCCGAGTCAAGACTGGTCGTAACGCTGCGTATCTTGTGGACGATGACGTTATGCAGCAGATAAAAGAGGGTGTCTTCCGTACTCGCGAAGAGATGGAAGAGTATCGcc                                                                                                                                                                                                                                                            <  1:196203/301‑1 (MQ=255)`  
                                                      `cTATTGGACACAATTGGTCGGTTCGCTAAGGCTGGTGCCGATATGTATACCGCTAAGGAACAACGAGCACGAGACCTAGCTGATGAACGCTCTAACGAGATTATCCGTAAGCTGACCCCTGAGCAACGTCGAGAAGCTCTCAACAACGGGACCCTTCTGTATCAGGATGACCCATACGCTATGGAAGCACTCCGAGTCAAGACTGGTCGTAACGCTGCGTATCTTGTGGACGATGACGTTATGCAGCAGATAAAAGAGGGTGTCTTCCGTACTCGCGAAGAGATGGAAGAGTATCGcatta                                                                                                                                                                                                                                                         >  1:148465/1‑297 (MQ=255)`  
                                                        `aTTGGACACCATTGGTCGGTTCGCTAAGGCTGGTGCCGATATGTATACCGCTAAGGAACAACGAGCACGAGACCTAGCTGATGAACGCTCTAACGAGATTATCCGTAAGCTGACCCCTGAGCAACGTCGAGAAGCTCTCAACAACGGGACCCTTCTGTATCAGGATGACCCATACGCTATGGAAGCACTCCGAGTCAAGACTGGTCGTAACGCTGCGTATCTTGTGGACGATGACGTTATGCAGCAGATAAAAGAGGGTGTCTTCCGTACTCGCCAAGAGATGGGAGAGTATCGccctgtc                                                                                                                                                                                                                                                       >  1:156172/1‑296 (MQ=255)`  
                                                         `ttGGACAGCATTGGTCGGTTCGCTAAGGCTGGTGCCGATATGTATGCCGCTAAGGAACAACGCGCACGAGACCTAGCTGATGAACGCTCTAACGAGATTATCCGTAAGCTGACCCCTGAGCAACGTCGAGAAGCTCTCAACAACGGGACCCTTCTGTATCAGGATGACCCATACGCTATGGAAGCACTCCGAGTCAATACTGGTCGTAACGCTGCGTATCTTGTGGACGATGACGTTATGCAGCAGATAAAAGAGGGTGTCTTCCGTACTCGCGAAGAGATGGAAGAGTATCGCCATAGTc                                                                                                                                                                                                                                                      <  1:293111/301‑1 (MQ=255)`  
                                                          `tGGACACCATTGGTCGGTTCGCGAAGGCTGGTGCCGATATGTATGCCGCTAAGGAACAACGAGCACGAGACCTAGCTGATGAACGCTCTAACGAGATTATCCGTAAGCTGACCCCTGAGCAACGTCGAGAAGCTCTCAACAACGGGACCCTTCTGTATCAGGATGACCCATACGCTATGGAAGCACTCCGAGTCAAGACTTGTCGTAACGCTGCGTATCTTGTGGACGATGACGTTATGCAGCAGATAAAAGAGGGTGTCTTCCGTACTCGCGAAGAGATGGAATAGTATCGCCATAGTCg                                                                                                                                                                                                                                                     <  1:421232/301‑1 (MQ=255)`  
                                                           `ggACACCATTGGTCGGTTCGCTAAGGCTGGTGCCGATATGTATACCGCTAAGGAACGACGAGCACGAGACCTAGCTGATGAACGCTCTAACGAGATTATCCGTAAGCTGACCCCTGAGCAACGTCGAGAAGCTCTCAACAACGGGACCCTTCTGTATCAGGATGACCAATACGCTATGGAAGCACTCCGAGTCAAGACTGGTCGTAACGCTGCGTATCTTGTGGACGATGACGTTATGCAGCAGATAAAAGAGGGTGTCTTCCGTACTCGCGAAGAGATGGAAGAGTATCGCCactgtctc                                                                                                                                                                                                                                                    >  1:67464/1‑294 (MQ=255)`  
                                                                `ccATTGGTCGGTTCGCTAAGGCTGGTGCCGATATGTATGCCGCTAAGGAACAACGAGCACGAGACCTAGCTGATGAACGCTCTAACGATATTATCCGTAAGCTGACCCCTGAGCAACGTCGAGAAGCTCTCAACAACAGGACCCTTCTGTATCAGGATGACCCATACGCTATGGAAGCACTCCGAGTCAAGACTGGTCGTAACGCTGCGTATCTTGTGGACGATGACGTTATGCAGCAGATAAAAGAGGGTGTCTACCGTACTCGCGAAGAGATGGAAGAGTATCGCCATAGTCGCCTTCa                                                                                                                                                                                                                                               >  1:237318/1‑301 (MQ=255)`  
                                                                `ccATTGGTCGGTTCGCTAAGGCTGGTGCCGATATGTATGCCGCTAAGGAACAACGAGCACGAGACCTAGCTGATGAACGCTCTAACGAGATTATCCGTAAGCTGACCCCTGAGCAACGTCGAGAAGCTCTCAACAACGGGACCCTTCTGTATCAGGATGACCCATACGCTATGTAAGCACTCCGAGTCAAGACTGGTCGTAACGCTGCGTATCTTGTGGACGATGACGTTATGCAGCAGATAAAAGAGGGTGTCTTCCGTACTCTCGAAGAGATGGAAGCGTATCGCCATAGTCGCCTTCa                                                                                                                                                                                                                                               >  1:33125/1‑301 (MQ=255)`  
                                                                `ccATTGGTCGGTTCGCTAAGGCTGGTGCCGATATGTATGCCGCTAAGGAACAACGAGCACGAGACCTAGCTGATGAACGCTCTAACGAGATTATCCGTAAGCTGACCCCTGAGCAACGTCGAGAAGCTCCCAACAACGGGACCCTTCTGTATCAGGATGACCCATACGCTATGGAAGCAATCCGAGACAAGACTGGTCGTAACGCCGCGTACCTTGTGGACGATGACGTTATGCAGCAGATAAAAGAGGGTGTATGCCGTACTCGCGAAGAGATGGAagctgtctcttatacacatctccg                                                                                                                                                                                                                                               >  1:416766/1‑279 (MQ=255)`  
                                                                `ccATTGGTCGGTTCGCTAAGGCTGGTGCCGATATGTATACCGCTAAGGAACAACGAGCACGAGACCTAGCTGATGAACGCTCTAACGAGATTATCCGTAAGCTGACCACTGAGCAACGTCGAGAAGCTCTCAACAACGGGACCCTTCTGTATCAGGATGACCCATACGCTATGGAAGCACTCCGAGTCAAGACTGGTCGTAACGCTGCGTATCTTGTGGACGATGACGTTATGCAGCAGATAAAAGAGGGTGTCTTCCGTACTCGCGAAGAGATGGAAGAGTATCGCCATAGTctgtctct                                                                                                                                                                                                                                               >  1:280736/1‑294 (MQ=255)`  
                                                                  `aTTGGTCGGTTCGCTAAGGGTGGGGCCGATAGGTCTACCGCTAAGGAACAACGAGCACGAGACCTAGCTGATGAACGCTCTAACGAGATTATCCGTAAGCTGAACCCTGAGCAACGTCGAGAAGCGCTCAACAACGGGACCCTTCTGTATCGTGATGACCCATACGCTATGGAAGCACTCCGAGTCAAGACTGGTCGTAACGCTGCGTATCTTGTGGACGATGACGTAATGCAGCAGATAAAAGAGGGTGTCTTCCGTACTCGCGAAGAGATGGAATAGTATCGCCATAGTCGCCTTCAag                                                                                                                                                                                                                                             <  1:349421/301‑1 (MQ=255)`  
                                                                                     `gtGGTGCCGATATGTATGCCGCTAAGGAACAACGAGCACGAGACCGAGCTGATGAACGCTCTAACGAGATTAGCCGTAAGCGGACCCCTGAGCAACGTCGAGAAGCTCTCAACAACGGGACCCTTCTATATCAGGATGACCCATACGCTATGGAAGCACTCCGAGTCAAGACTGGTCGTAACGCTGCGTATCTTGTGGACGATGACGTTATGAAGCAGATAAAAGAGGGTGTCTTCCGTACTCGCGAAGAGATGGAAGAGTATCGCCATAGTCGCCTTCAAGAGGGCGCTAAGGTATACGc                                                                                                                                                                                                                          <  1:17651/300‑1 (MQ=255)`  
                                                                                     `gctcggagatgtgtataagagacagggAACAACGAGCACGAGACCTAGCTGATGAACGCTCTAACGAGATTATCCGTAAGCTGACCCCTGAGCAACGTCGAGAAGCTCTCAACAACGGGACCCTTCTGTATCAGGATGACCCATACGCTATGGAAGCACTCCGAGTCAAGACTGGTCGTAACGCTGCGTATCTTGTGGACGATGACGTTATGCAGCAGATAAAAGAGGGTGTCTTCCGTACTCGCGAATAGATGGAAGAGTATCGCCATAGTCGCCTTCAAGAGGGCGCTAAGGTATACGc                                                                                                                                                                                                                          <  1:80312/276‑1 (MQ=255)`  
                                                                                     `agagacaggATATGTAAACCGCTAAGCAACAACGAGCACGAGACCTAGCGGATGAACGCTCTAACGAGATTATCCGTAAGCTGACCCCTGAGCAACGTCGAGAAGCTCTCAACACCGGGACCCTTCTGTATCAGGATGACCCATACGCTATGGAAGCACTCCGAGTCAAGACTGGTCGTAACGCTGCGTATCTTGTGGACGATGACGTTATGCAGCAGATAAAAGAGGGTGTCTTCCGTACTCGCGAAGAGATGGAAGAGTATCGCCAAAGTCGCCTTCAAGAGGGAGCTAAGGTATACGc                                                                                                                                                                                                                          <  1:443239/293‑1 (MQ=255)`  
                                                                                                `atGTATACCGCTAATGAACAACGAGCACGAGACCTAGCTGATGAACGCTCTAACGAGATTATCCGTAAGCTGACCCCTGAGCAACGTCGAGCAGTTCTCAACAACGGGACCCTTCTGTATCAGGATGACCCATACGCTATGGAAGCACTCCGAGTCAAGACTGGTCGTAACGCTGCGTATCTTGTGGACGATGTAGTTATGCAGCAGATAAAAGAGGGTGTCTTCCGTACTCGCGCAGAGATGGAAGACTATCGCTATAGTCGCATCTAAGAGGCCACTAAGGTATACggctgcttccttt                                                                                                                                                                                                               >  1:492052/1‑289 (MQ=255)`  
                                                                                                 `tGTATGCCGCTAAGGAACAACGAGCACGAGACCTAGCTGATGAACGCTCTAACGAGATTATCCGTAAGCTGACCCCTGAGCAACGTCGAGAAGCTCACAACAACGGGACCCTTCTGTATCAGGATGACCCATACGCTATGGAAGCACTCCGAGTCAAGACTGGTCGTAACGCTGCGTATCTTGTGGACGATGACGTTATGCAGCAGATAAAAGAGGGTGTCTTCCGTACTCGCGAAGAGATGGAAGAGTATCGCCATAGTCGCCTTCAAGAGGGCGCTAAggctgtctcttatacacatct                                                                                                                                                                                                              >  1:223955/1‑282 (MQ=255)`  
                                                                                                  `gtatgacGCTAAGGAACAACGAGCACGAGACCTAGCTGATGAACGCTCTAACGAGATTATCCGTAAGCTGACCCCTGAGCAACGTCGAGAAGCTCTCAACAACGGGACCCTTCTGTATCAGGATGACCCATAAGCTATGGAAGCACTCCGAGTAAAGACTGGTCGTAACGCTGCGTATCGTGTGGACGATGACGTTATGCAGCAGATAAAAGAGGGTGTCTTCCGTACTCGCGAAGAGATGGAAGAGTATCGCCATAGTCGCCTTCAAGAGGGCGCTAAGGTATACGCTGAGCAGTTCGGc                                                                                                                                                                                                             <  1:20153/295‑1 (MQ=255)`  
                                                                                                  `gTATACCGCTAAGGAACAACGAGCACGAGACCTAGCTGATGAACGCTCTAACGAGATTATCCGTAAGCTGACCCCTGAGCAACGTCGAGAAGCTCTCAACAACGGGACCCTTCTGTATCAGGATGACCCATACGCTATGGAAGCACTCCGAGTCAAGACTGGTCGTAACGCTGCGTATCTTGTGGACGATGACGTTATGCAGCAGATAAAAGAGGGTGTCTTCCGTACTCGCGAAGAGATGGAAGAGTATCGCCATAGTCGCCTTCAAGAGGGCGCTAAGGTATACGcctgtctcttatac                                                                                                                                                                                                             >  1:248375/1‑288 (MQ=255)`  
                                                                                                       `ccGCTAAGGAACAACGAGCACGAGACCTAGCTGATGAACGCTCTAACGAGATTATCCATAAGCTGACCCCTGAGCAACGTCGAGAAGCTCTCATCAACGGGACCCTTCTGTATCAGGATGACCCATACGCTATGGAAGCACTCCGAGTCAAGACTGGTCGTAACGCTGCGTATCTTGTGGACGATGACGTTATGCAGCAGATAAAAGAAGGTGTCTTCCGTACTCGCGAAAGAATGGAAGAGTATGTCCATAGTCCCCTTCAAGAGGGCGCTAGGGTATACGCTGAGCCGTTAGGAATCGa                                                                                                                                                                                                        >  1:353386/1‑301 (MQ=255)`  
                                                                                                        `cGCTAAGGAACAACGAGCACGAGACCTAGCTGATGAACGCTCTAACGAGATTATCCGTAAGCTGACCCCTGAGCAACGTCGAGAAGCTCTCAACAACGGGACCCTTCTGTATCAGGATGACCCATACGCTATGGAAGCACTCCGAGTCAAGACTGGTCGTAACGCTGCGTATCTTGTGGACGATGACGTTATGCAGCAGATAAAAGAGGGTGTCTTCCGTACTCGCGAAGAGATGGAAGAGTATCGCCATAGTCGCCTTCAAGAGGGCGCTAAGGTATACGCTGAGCAGTTCGGCATCGAc                                                                                                                                                                                                       <  1:206188/301‑1 (MQ=255)`  
                                                                                                            `gtatggttttaagagacagaCCTGGCTGATGAACGCTCGAACGAGATTATACGTAAGCTGACCCCGGAGCAACGTCGAGAAGCTCTCAACAACGGGCCCCTTCTGTATCAGGATGACCCATACGCTATGGAAGCACTCCGAGTCAAGACTGGTCGTAACGCTGCGTATCTTGTGGACGATGACGTTATGCAGCAGATAAAAGAGGGTGTCTTCCGTACTCGCGAAGAGATGGAAGAGTATCGCCATAGTCGCCTTCAAGAGGGCGCTAAGGTATACGCTGAGCAGTTCGGCATCGACCCTg                                                                                                                                                                                                   <  1:319746/284‑1 (MQ=255)`  
                                                                                                               `gAACAACGAGCACGAGACCTAGCTGATGAGCGCTCTAACGAGATTATCCGTAAGCTGACCCCTGAGCAACGTCGGGAAGCTCTCAACAACGGGACCCTTCTGTATCAGGATGACCCATACGCTATGGAAGCACTCCGAGTCAAGACTGGTCGTAACGCTGCTTATCTTGTGGACGATGACGTTATGCAGCATATAAAAGAGGGTGTCTTCCGTACTCGCGAAGAGATGGAAGAGTATCGCCATAGTCGCCTTCAAGAGGGCGCTAAGGTATACGCTGAGCAGTTCGGCATCGACCCTGAgg                                                                                                                                                                                                <  1:91612/301‑1 (MQ=255)`  
                                                                                                               `gAACAACGAGCACGAGACCTAGCTGATGAACGCTCTAACGAGATTATCCGTAAGCTGACCCCTGAGCAACGTCGAGAAGCTCTCAACAACGGGACCCTTCTGTATCAGGATGACCCATACGCTATGGAAGCACTCCGAGTCAAGACTGGTCGTAACGCTGCGTCTCTTGTGGACGATGACGTTATGCAGCAGATAAAAGAGGGTGTCTTCCGTACTCCCGAAGAGATGGAAGAGTATCGCCATAGTCGCCTTCAAGAGGGCGCTAAGGTATACGCTGAGCAGTTCGGCATCGACCCTGAgg                                                                                                                                                                                                >  1:36714/1‑301 (MQ=255)`  
                                                                                                                      `gAGCACGAGACCTAGCTTATGACCGCTCTAACGAGATTATCCGTAAGCTGACCCCTGAGCAACGTCGAGAAGCTCTCAACAACGGGACCCTTCTGTATCAGGATGACCCATACGCTATGGAAGCACTCCGAGACAAGACTGGTCGTAACGCTGCGTATCTTGTGGACGATGACGTTATGCAGCAGATAAAAGAGGGTGTCTTCCGTACTCGCGAAGAGATGGAAGAGTATCGCCATAGTCGCCTTCAAGAGGGCGCTAAGGTATACGCTGAGCAGTTCGGCATCGACCCTGAGGACGTTGa                                                                                                                                                                                         <  1:299/301‑1 (MQ=255)`  
                                                                                                                             `agaCCTAGCTGATGAACGCTCTAACGAGATTATCCGTAAGCTGACCCCTGAGCAACGTCGAGAAGCTCTCAACAACGGGACCCTTCTGTATCAGGATGACCCATACGCTATGGAAGCACTCCGAGTCAAGACTGGTCGTAACGCTGCGTATCTTGTGGACGATGACGTTATGCAGCAGATAAAAGATGGTGTCTTCCGTACTCGCGAAGAGATGGAAGAGTATCGCCATAGTCGCCTTCAAGAGGGCGCTACGGTATACGCTGAGCCGTTCGGCATCGACCCTGAGGACGTTGATtctctt                                                                                                                                                                                  >  1:389673/1‑299 (MQ=255)`  
                                                                                                                                      `tgatgaACGCTCTAACGAGATTATCCGTAAGCTGACCACTGAGCAACGTCGAGAAGCTCTCAACAACGGGACCCTTCTGTATCAGGATGACCCATACGCTATGGAAGCACTCCGAGTCAAGACTGGTCGTAACGCTGCGTATCTTGTGGACGATGACGTTATGCAGCAGATAAAAGAGGGTGTCTTCCGTACTCGCGAAGAGATGGAAGAGTATCGCCATAGTCGCCTTCAAGAGGGCGCTAAGGTATACGCTGAGCAGTTCGGCATCGACCCTGAGGACGTTGATTATCAGCATGGTTTc                                                                                                                                                                         <  1:462203/301‑1 (MQ=255)`  
                                                                                                                                              `gCTCTAACGAGATTATCCGGAAGCTGACCCCTGAGCAACGTCGAGAAGCTCTCAACAACGTGACCCTTCTGTATCAGGATGACCCATACGCTATGGAAGCACTCCGAGTCAAGACTGGTCTTAACGCTGCGTATCTTGTGGACGATGACGTTATGCAGCAGATAAAAGAGGGTGTCTTCCGTACTCGCGAAGAGATGGAAGAGTATCGCCATAGTCGCCTTCAAGAGGGCGCTAAGGTATACGCTGAGCAGTTCGGCATCGACCCTGAGGACGTTGATTATCAGCGTGGTTTCAACGGGGa                                                                                                                                                                 <  1:327468/301‑1 (MQ=255)`  
                                                                                                                                                     `cGAGATTATCCGTAAGCTGACCCCTGAGCAACGTCGAGAAGCTCTAAACAACGGGACCCTTCTGTATCAGGATGACCCGTACGCTATGGAAGCACTCCGAGTCAAGACTGGTCGTAACGCTGCGTATCTTGTGGACGATGACGTTATGCAGCAGATAACAGAGGGTGTCTTCCGTACTCGCGAAGAGATGGAAGAGTATCGCCCTAGTCGCCTTCAAGAGGGCGCTAAGGTATACGCTGAGCAGTTCGGCATCGACCCTGAGGCCGTTGATTATCAGCGTGGTTTCAACGGGGACATTAcc                                                                                                                                                          >  1:214756/1‑301 (MQ=255)`  
                                                                                                                                                             `tCCGTAAGCTGACCCCTGAGCAACGTCGAGAAGCTCTCAACAACGGGACCCTTCTGTATCAGGATGACCCATACGCTATGGAAGCACTCCGAGTCAAGACTGGTCGTAACGCTGCGTATCTTGTGGACGATGACGTTATGCAGCAGATAAAAGAGGGTGTCTTCCGTACTCGCGAAGAGATGGAAGAGTATCGACATAGTCGCCTTCAAGAGGGCGCTAAGGTATACGCTGAGCAGTTCGGCATCGACCCTGAGGACGTTGATTATCAGCGTGGTTTCAACggggcatttacgccgtccct                                                                                                                                                  >  1:138047/1‑285 (MQ=255)`  
                                                                                                                                                              `ccGGAAGCTGACCCCTGAGTAACGTCGAGAAGCTCTCAACAACGGGACCCTTCTGTATCAGGATGACCCATACGCTATGGAAGCACTCCGAGCCAAGACTGGTCGTAACGCTGCGTATCTTGTGGACGATGACGGTATGCAGCAGATAAAAGAGGGTGTCTTCCGTACTCGCGAAGAGATGGAAGAGTATCGCCATAGTCGCCTTCAAGAGGGCGCTAAGGTATACGCTGAGCAGTTCGGCATCGACCCTGAGGACGTTGATTATCAGCGTGGTTTCAACGGGGACATTACCGAGCGTAAc                                                                                                                                                 <  1:48005/301‑1 (MQ=255)`  
                                                                                                                                                                `gaaaGCTGACCCCTGGGCAACGTCGAGAAGCTCTCAACAACGGGACCCTTCTGTATCAGGATGACCCATACGCTATGGAAGCGCTCCGAGTCAAGACTGGTCGTAACGCTGCGTATCTTGTGGACGATGACGTTATGCAGCAGATAAAAGAGGGTGTCTTCCGTACTCGCGAAGAGATGGAAGAGTATCGCCATAGTCGCCTTCAAGAGGGCGCTAAGGTATACGCTGAGCAGTTCGGCATCGACCCTGAGGACGTTGATTATCAGCGTGGTTTCAACGGGGACATTACCGAGCGTAACAt                                                                                                                                               <  1:272050/299‑1 (MQ=255)`  
                                                                                                                                                                       `agccccTGAGCAACGTCGAGAATATCTCAACAACGGGACCCTTCTGTATCAGGATTACCCTTACGCTATGGAAGCACTCCGAGTCAAGACTGGTCGTAACGCTGCGTATCTTGTGGACGATGACGTTATGCAGCAGATAAAAGAGGGTGTCTTCCGTACTCGCGAAGAGATGGAAGAGTATCGCCATAGTCGCCTTCAAGAGGGCGCTAAGGTATACGCTGAGCAGTTCGGCATCGACCCTGAGGACGTTGATTATCAGCGTGGTTTCAACGGGGACATTACCGAGCGTAACATCTCGCTg                                                                                                                                        <  1:43198/299‑1 (MQ=255)`  
                                                                                                                                                                         `ccccTGAGCAACGTCGAGAAGCTCTCAACAACGGGACCCTTCTGTATCAGGATGACCCATACGCTATGGAAGCACTCCGAGTCAAGACTGGTCGTAACGCTGCGTATCTTGTGGACGATGACGTTATGCAGCAGATAAAAGAGGGTGTCTTCCGTACTCGTGAAGAGATGGAAGAGTATCGCCATAGTCGCCTTCAAGAGGGCGCTAAGGTATACGCTGAGCAGTTCGGCATCGACCCTGAGGACGTTGATTATCAGCGTGGTTTCAACGGGGACATTACCGAGCGTAACATCTCGCTGTa                                                                                                                                      <  1:252583/301‑1 (MQ=255)`  
                                                                                                                                                                         `ccccTGAGCAACGTCGAGAAGCTCTCAACAACGGGACCCTTCTGTATCAGGATGACCCATACGCTATGGAAGCACTCCGAGTCAAGACTGGTCGTAACGCTGCGTATCTTGTGGACGATGACGTTATGCAGCAGATAAAAGAGGGTGTCTTCCGTACTCGCGAAGAGATGGAAGAGTATCGCCATAGTCGCCTTCAAGAGGGCGCTAAGGTATAAGCTGAGCAGTTCGGCATCGACCCTGAGGACGTTGATTATCAGCGTGGTTTCAACGGGGACATTACCGAGCGTAACAtctgtctctt                                                                                                                                      >  1:151926/1‑294 (MQ=255)`  
                                                                                                                                                                          `acaggAGGAACGTCGGGAAGCTCTCAACAACGGGACACTTCTGTATCAGGATGAACCATACGCTATGGAAGCACTCCGAGTCAAGACTGGTCGTAAAGCTGCGTATCTTGTGGACGATGACGTTATGCAGCAGATAAAAGAGGGTGTCTTCCGTACTCGCGAAGAGATGGAAGAGTATCGCCATAGTCGCCTTCAAGAGGGCGCTAAGGTATACGCTGAGCAGTTCGGCATCGACACTGAGGACGTTGATTATCAGCGTGGTTTCAACGGGGACATTACCGAGCGTAACATCTCGCTGTAt                                                                                                                                     <  1:143716/297‑1 (MQ=255)`  
                                                                                                                                                                            `cTGAGGAACGTCGAGAAGCTCTCAACAAGGGGACCCTTCTGTATCAGGATGACCCATACGATATGGAAGCACTCCGAGTCAAGACTGGTCGTAACGCTGCGTATCTTGTGGACGATGACGTTATGCAGCAGATAAAAGAGGGTGTCTTCCGTACTCGCGAAGAGATGGAAGAGTATCGCCATAGTCGCCTTCAAGAGGGCGCTAAGGTATACGCTGAGCAGTTCGGCATCGACCCTGAGGACGTTGATTATCAGCGTGGTTTCAACGGGGACATTACCGAGCGTAACATCTCGCTGTATgg                                                                                                                                   <  1:133833/301‑1 (MQ=255)`  
                                                                                                                                                                            `atgtgtataagagacaggCTCTCAACAACGGGACCCGTCTGTATCAGGATGACCCATACGCTATGGAAGCACTCCGAGTAAAGACTGGTCGTAACGCTGCGTATCTTGTGGACGAGGACGTTATGCAGCAGATAAAAGAGGGTGTCTTCCGTACTCGCGAAGAGATGGAAGAGTATCGCCATAGTCGCCTTCAAGAGGGCGCTAAGGTATACGCTGAGCAGTTCGGCATCGACCCTGAGGACGTTGATTATCAGCGTGGTTTCAACGGGGACATTACCGAGCGTAACATCTCGCTGTATgg                                                                                                                                   <  1:405898/284‑1 (MQ=255)`  
                                                                                                                                                                               `aGCAAGGTCTAGAAGCTGTCAACAACGGGACCCTTCTGTATCAGGATGACCAATAAGGTATGGAAGCACTCCGAGTCAAGACTGGTCGTAACGCTGCGTATCTGGTGGACGATGACGTTATGCAGCAGATAAAAGAGGGTGTCTTCCGTACGCGCGAAGAGATGGAAGAGTATCGCCATAGTCGCCTTCAAGAGGGCGCTAAGGTATACGCTGAGCAGTTCGGCATCGACCCTGAGGACGTTGATTATCAGCGTGGTTTCAACGGGGACATTACCGAGCGTAACATCTCGATGTATGGTgc                                                                                                                                <  1:362850/301‑1 (MQ=255)`  
                                                                                                                                                                                 `aaaCGTCGAGAAGCTCTCAACAACGGGACCCTTCTGTATCAGGATTACCCATACGCTATGGAAGCACTCCGAGTCAAGACTGGTCGTAACGCTGCGTATCTTGTGGACGATGACGTTATGCAGCAGATAAAAGAGGGTGTCTTCCGTACTCGCGAAGAGATGGAAGAGTATCGCCATAGTCGCCTTCAAGAGGGCGCTAAGGTATACGCTGAGCAGTTCGGCATCGACCCTGAGGACGTTGATTATCAGCGTGGTTTCAACGGGGACATTACCGAGCGTAACATCTCGCTGTATGGTgcgc                                                                                                                              <  1:385604/300‑1 (MQ=255)`  
                                                                                                                                                                                     `gTCGAGAAGCTCTCAACAACGGGACCCTTCTGTATCAGGATGACCCATACGCTATGGAAGCACTCCGAGTCAAGACTGGTCGTAACGCTGCGTATCTTGTGGACGATGACGTTATGCAGCAGATAAAAGAGGGTGTCTTCCGTACTCTCGAAGAGATGGAAGAGTATCGCCATAGTCTCCTTCAAGAGGGCGCTAAGGTATACGCTGAGCAGTTCGGCATCCAACCTAAGGACGTTGATTATCAGCGTGGTTTCAACGGGGACATTACCGAGCGTAAACACTCGCTGTATgactgcttctt                                                                                                                          >  1:313043/1‑291 (MQ=255)`  
                                                                                                                                                                                         `agaAGCTCTCAACAACGGGACCCTTCTGTATCAGGATGACCCATACGCTATGGAAGCACTCCGAGTCAAGACTGGTCGTAACGCTGCGTATCTTGTGGACGATGACGTTATGCAGCAGATAAAAGAGGGTGTCTTCCGTACTCGCGAAGAGATGGAAGAGTATCGCCATAGTCGCCTTCAAGAGGGCGCTAAGGTATACGCTGAGCAGTTCGGCATCGACCCTGAGGACGTTGATTATCAGCGTGGTTTCAACGGGGACATTACCGAGCGTAACATCTCGCTGTATGGTGCGAATGATAAc                                                                                                                      <  1:323438/301‑1 (MQ=255)`  
                                                                                                                                                                                           `aaGCTCTCAAAAACGGGACGCTTCTGTATCAGGATGACCAATACGCTATGGAAGCACTCCGAGTCAAGACTGGTCGTAACGCTGCGTATCTTGTGGACGATGACGTTATGCAGCAGATAAAAGAGGGTGTCTTCCGTACTCGCGAAGAGATGGAAGAGTATCGCCATAGTCGCCTTCAAGAGGGCGCTAAGGTATACGCTGAGCAGTTCGGCATCGACCCTGAGGACGTTGATTATCAGCGTGGTTTCAACGGGGACATTACCGAGCGTAACATCTCGCTGTATGGTGCGCATGATAActt                                                                                                                    <  1:454234/301‑1 (MQ=255)`  
                                                                                                                                                                                                `ctcAACAACGGGACCCTTCTGTATCAGGATGACCCATACGCTATGGAAGCACTCCGAGTCAAGACTGGTCGTAACGCTGCGTATCTTGTGGACGATGACGTTATGCAGCAGATAAAAGAGGGTGTCTTCCGTACTCGCGACGAGATGGAAGAGTATCGCCATAGTCGCCTTCAAGAGGGCGCTAATGTATACGCTGAGCAGTTCGGCATCGACCCTGAGGACGTTGATTATCCGCGTGGTTTCAACGGGGACATTACCGAGCGTAACATCTCGCTGTATGGTTCGCCTGCTAACTTCTTGa                                                                                                               >  1:211391/1‑301 (MQ=255)`  
                                                                                                                                                                                                   `aacaacGGGACCCTTCGGTATCAGGATGACCCATACGCTATGGAAGCACTCCGAGTCAAGACTGGTCGTAACGCTGCGTATCTTGTGGACGATGACGTTATGCAGCAGATAAAAGGGGGTGTCTACCGTACTCGCGAAGAGATGGAAGAGTATCGCCATAGTCGCCTTCAAGAGGGCGCTAAGGTATACGCTGAGCAGTTCGGCATCGACCCTGAGGACGTTGATTATCAGCGTGGTTTCAACGGGGACATTACCGAGCGTAACATCTCGCTGTATGGTGCGCATGATAACTTCTTGAGcc                                                                                                            <  1:172336/301‑1 (MQ=255)`  
                                                                                                                                                                                                     `caacGGGACCCTTCTGTATCAGGATGACCCATACGCTATGGAAGCACTCCGAGTCAAGACTGGTCGTAACGCTGCGTATCTTGTGGACGATGACGTTATGCAGCAGATAAAAGAGGGTGTCTTCCGTACTCGCGAAGAGATGGAAGAGTATCGCCATAGTCGCCTTCAAGAGGGCGCTAAGGTATACGCTGAGCAGTTCGGCATCGACCCTGATGACGTTGATTATCAGCGTGGTTTCAACGGGGACATTACCGAGCGTAACATCTCGCTGTATGGTGCGCATGATAActtctgtctcttt                                                                                                          >  1:483912/1‑293 (MQ=255)`  
                                                                                                                                                                                                            `aCCCTTCTGTATCAGGATGACCCATACGCTATGGAAGCACTCCGAGTCAAGACTGGTCGTAACGCTGCGTATCTTGTGTACGATGACGTTATGCAGCAGATAAAAGAGGGTATCTTCCGTACTCGCGAAGAGATGGAAGAGTATCGCCATAGTCGCCTTCAAGAGGGCGCTAAGGTATACGCTGAGCAGTTCGGCATCGACCCTGAGGACGTTGATTATCAGCGTGGTTTCAACGGGGACATTACCGAGCGTAACATCTCGCTGTATGGTGCGCATGATAACTTCTTGAGCCAGCAAGCTc                                                                                                   <  1:266327/301‑1 (MQ=255)`  
                                                                                                                                                                                                               `cTTCTGTATCAGGATGACCCATACGCTATGGAAGCACTCCGAGTCAAGACTGGTCGTAACGCTGCGTATCTTGTGGACGATGACGTTATGCAGCAGATAAAAGAGGGTGTCTTCCGTACTCGCGAAGAGATGGAAGAGTATCGCCATAGTCGCCTTCAAGAGGGCGCTAAGGTATACGCTGAGCAGTTCGGCATCGACCCTGAAGACGTTGATTATCAGCGTGGTTTCAACGGGAACATTACCGAGCGTAACATCTCGCTGTATGGTGCGCactgtctcttatacacatctccgcgcccac                                                                                                >  1:340565/1‑272 (MQ=255)`  
                                                                                                                                                                                                               `cTTCTGTATCAGGATGACCCATACGCTATGGAAGCACTCCGAGTCAAGACTGGTCGTAACGCTGCGTATCTTGTGGACGATGACGTTATGCAGCAGATAAAAGAGGGTGTCTTCCGTACTCGCGAAGAGATGGAAGAGTATCGCCATAGTCGCCTTCAAGAGGGCGCTAAGGTATACGCTGAGAAGTTCGGCATCGACCCTGAGGACGTTGATTATCAGCGTGGTTTCAACGGGGACATTACCGAGCGTAACATCTGGCTGTATGGTGCGCATGATAAATGCTTGAGCCatgtctcttata                                                                                                >  1:95834/1‑290 (MQ=255)`  
                                                                                                                                                                                                                `ttCTGTATCAGGATGACCCATACGCTATGGAAGTACTCCGAGTCCAGACTGGTCGTAACGCTGCGTATCTTGTGTACGATGACGTTATGCAGCAGATAAAAGAGGGTGTCTTCCGTACTCGCGAAGAGATGGAAGAGTATCGCCATAGTCGCCTTCAAGAGGGCGCTTAGGTATCAGCTGCGCAGTTCGGCATCGTACCTGAGGACGTTGATTATCCGAGTGGTTTAAACGGGGACATTACAGAGCGTAACATCTCGCTGTATGGTGCGCATGATAACTTATTGCGCCAGCACGATCAGaa                                                                                               >  1:192243/1‑301 (MQ=255)`  
                                                                                                                                                                                                                `ttCTGTATCAGGATGACCCATACGATATGGAAGCACTCCGAGTCAAGACTGGTCGTAACGCTGCGTATCTTGTGGACGATGACGTTATGCAGCAGATAAAAGAGGGTATCTTCCGTACTCGCCACGAGATGGAAGATTATCGCCATAGTAGCCTTCAAGTCGGCGCTAAGGTTTACGCTGAGCAGTTCGGCATCGACCCTGAGGACGTTGTTTATCAGCGTGGTTTCAACGGGGTCATTACCCAGCTTAACATCTCGCTGTATGGTGCGCATGATACcttctctgtctcttctacacatct                                                                                               >  1:51259/1‑282 (MQ=255)`  
                                                                                                                                                                                                                 `tatGTATCAGGATAACCCATACGCTATGTAAGCACACCGAGTCAAAACAGGTCGTAACGCTCCGTATCCTGTGAACAAGTCCGTTAAGCCACAGATAAAAGAGGATTTCTGCCGTACTCGCGAAGAAATGGAAGACTATCGCCATAGTCGCCTTCAAGATCTCGCTAAGATATACGCTGAGCAGTTCGGCATCGACCCTGAGAACGTTGATTATCAGCTTGGTTTCAACGAGGACATTACCGAGCGTAACATCTCGCTGTATGGTGCGCATGATAACTTCTTGAGCCAGCAAGCTCAGAAg                                                                                              <  1:44853/299‑1 (MQ=255)`  
                                                                                                                                                                                                                 `tCTGTATCAGGATGACCCATACGCTATGGAAGCACTCCGAGTCAAGACTGGTCGTAACGCTGCGTATCTTGTGGACGATGACGTTATGCAGCAGATAAAAGAGGGTGTCTTCCGTACTCGCGAAGAGATGGAAGAGTATCGCCATAGTCGCCTTCAAGAGGGCGCTAAGGTATACGCTGAGCAGTTCGGCATCGACCCTGAGGACGTTGATTATCAGCGTGGTTTCAACGGGGACATTACCGAGCGTAACATCTCGCTGTATGGTGCGCATGATAACTTCTTGAGCCAGCAAGCTCAGAAg                                                                                              <  1:181732/301‑1 (MQ=255)`  
                                                                                                                                                                                                                 `tCTGCAACAGTATGACCCAAATGGTATGGAAGCAGTGCGAGACAAGACTGGTCGTAACGCTGAGTATATTGTGGACGATGACGTTATGCAGCAGATAAAAGAGGGTGTCTTCCGTACTCGCGAAGAGATGGAAGAGTATCGCCATAGTCGCCTTCAAGAGGGCGAAAAGGTATAAGCTGAGCAGTTCGGCATCGACCCTGAGGACGTTGATTATCAGCGTGGTTTCAACGGGGACATTACAGAGCGGAACATCTCGCTGTATGGTGCGCATGATAACTTCTTGAGCCAGCAAGCTCAGAAg                                                                                              <  1:488716/301‑1 (MQ=255)`  
                                                                                                                                                                                                                  `cTGTATCAGGATGACCCATACGCTATGGAAGCACTCCGAGTCAAGACTGGTCGTAACGCTGCGTATCTTGTGGACGATGACGTTATGCCGCAGATAAAAGAGGGTGTCTTCCGTACTCGCGAAGAGATGGAAGAGTATCGCCATAGTCGCCTTCAAGAGGGCGCTAAGGTATACGCTGAGCAGTTCGGCATCGACCCTGAGGACGTTGATTATCAGCGTGGTTTCAACGGGGACATTACCGAGCGTACCATCTCGCTGTATGGTGCGCATGATCACTTCTTGAGCCAGCAAGCTCAGAAgg                                                                                             >  1:401620/1‑301 (MQ=255)`  
                                                                                                                                                                                                                    `gTATCAGGATGACCCATACGCTATGGAAGCACTCCGAGTCAAGACTGGTCGTAACGCTGCGTATCTTGTGGACGATGACGTTATGCAGCAGATAAAAGAGGGTGTCTTCCGTACTCGCGAAGAGATGGAAGAGTATCGCCATAGTCGCCTTCAAGAGGGCGCTAAGGTATACGCTGAGCAGTTCGGCATCGACCCTGAGGACGTTGATTATCAGCGTGGTTTCAACGGGGACATTACCGAGCGTAACATCTCGCTGTATGGTGCGCATGATAACTTCTTGAGCCAGCAAGCTCAGCAgggg                                                                                           >  1:169304/1‑300 (MQ=255)`  
                                                                                                                                                                                                                    `gTATCAGGATGACCCATACGCTATGGAAGCACTCCGAGTCAAGACTGGTCGTAACGCTACGTATCTTGTGGACGATGACGTTATGCAGCAGATAAAAGATGGTGTCTTCCGTACTCGCGAAGAGCTGGAAGAGTATCGCCATAGTCGCCTTCAAGAGAGCGCTAAGGTATACGCTGAGCAGTTCGGCATCGACCCTGAGGACGTTGATTATACGCGTGGTTTCAACGGGGACATTACCGAGCGTAACATGTCGCTGTATGGTGCGCATGATAACTTCTTGAGCCAGCACGCTCAGAATGgc                                                                                           >  1:61332/1‑301 (MQ=255)`  
                                                                                                                                                                                                                     `aagagacaggaaccATACGCTAGGGAAGCACTCCGAGTCAAGACTGGTCGTAACGCTGCGTATCTTGTGGACGATGACGTTATGCAGCAGATAAAAGAGGGTGTCTTCCGTACTCGCGAAGAGATGGAAGAGTATCGCCATAGTCGCCTTCAAGAGGGCGCTAAGGTATACGCTGAGCAGTTCGGCATCGACCCTGAGGACGTTGATTATCAGCGTGGTTTCAACGGGGACATTACCGAGCGTAACATCTCGCTGTATGGTGCGCATGATAACTTCTTGAGCCAGCAAGCTCAGAAGGgcg                                                                                          <  1:393877/289‑1 (MQ=255)`  
                                                                                                                                                                                                                              `gACCCATACGCTATGGAAGCACTCCGAGTCAAGACTGGTCGTACCGCTGCGTATCTTGTGGACGATGACGTTATGCAGCAGATAAAAGAGGGTGTCTTACGTAATCGCGAAGAGATGGAAGAGTAACGCCATAGTCGCCTTCACAAGGGCGCTAAGGTAGACGCTGAGCAGTTCGGAATCGACCATGAGGACGTTTCTTATCAGAGTGGTTTTACAGTGGTCATTACAGAGCGTAAGATCTCGCTGTATAGTGAGCATGATCACGTCTGACGCCAGAAGGCACAGATGGGAGGTATGAtaa                                                                                 >  1:244774/1‑299 (MQ=255)`  
                                                                                                                                                                                                                                `cccATACGCTATGGAAGCACTCCGAGTCAAGACTGGTCGTAACGCTGCGTATCTTGTGGACGATGACGTTATGCAGCAGATAAAAGAGGGTGTCTTCCGTACTCGCGAAGAGATGGAAGAGTATCGCCATAGTCGCCTTCAAGAGGGCGCTAAGGTATACGCTGAGCAGTTCGGCATCGACCCTGAGGACGTTGATTATCAGCGTGGTTTCAACGGGGACATTACCGAGCGTAACATCTCGCTGTATGGTGCGCATGATAACTTCTTGAGCAAGCAAGCTCAGAAGGGCGCTATCATGAAc                                                                               <  1:277151/301‑1 (MQ=255)`  
                                                                                                                                                                                                                                `cccATACGCTATGGAAGCACTCCGAGTCAAGACTGGTCGTAACGCTGCGTATCTTGTGGACGATGACGTTATGCAGCAGATAAAAGAGGGTGTCGTCCGTACTCGCGAAGAGATGGAAGAGTATCGCCATAGTCGCCTTCAAGAGGGCGCTAAGGTATACGCTGAGCAGTTCGGCATCGACCCTGAGGACGTTGATTATCAGCGTGGTTTCAACGGGGACATTACCGAGCGTAACATCTCGCTGTATGGTGCGCATGATAACTTCTTGAGCCAGCAAGCTCAGAAGGGCGCTATCATGAAc                                                                               <  1:474736/301‑1 (MQ=255)`  
                                                                                                                                                                                                                                `cccATACGCTATGGAAACACTCCGAGTCAAGCCTGGTCCTAACGCGTCGTATCTTGTGGACTATGACTTTATGCAGCAGATAAAAGAGGGTGTCTTCCGTACTCGCGAAGAGATTGACGAGTATCGCCATAGTCGCCTTCAAGAGGGCTCTAAGGTATACGCTGAGCAGTTCGGCATCGACCCTGAGGACGTTGATTATCCGCATGGTTTCAACGGGGACATTACCGAGCGTAACATCTCGCTGTATGGTGCGCATGATAACTTCTTGAGCCAGCAAGCTCAGAAGGGCGCTATCATGAAc                                                                               <  1:129573/301‑1 (MQ=255)`  
                                                                                                                                                                                                                                        `cTATGGAAGCACTCCGAGTAAAGACTGGTCGTAATGCTGCGTATCTTGGGGACGATGACGTTAGGCAGCAGATAAAAGATGGTGTCTTCCGTACTCGCGAAGAGATGGAAGAGTATCGCCATAGTCGCCTTCAAGAGGGCGCTAAGGTATACGCTGAGCAGTTCGGCATCGACCCTGAGGACGTTGATTATCAGCGTGGTTTCAACGGGGACATTACCGAGCGTAACATCTCGCTGTATGGTGCGCATGATAACTTCTTGAGCCAGCAAGCTCAGAAGGGCGCTATCATGAACAGCCGAGt                                                                       <  1:57125/301‑1 (MQ=255)`  
                                                                                                                                                                                                                                               `tgtataagagacagaGACTGGTCGTAAGGCTGCGTATCTTGTGGACGATGACGTTATGCAGCAGATAAAAGAGGGTGTCTTCCGTACTAGCGAAGAGATGGAAGAGTATCGCCATAGTCGCCTTCAAGAGGGCGCTAAGGTATACGCTGAGCAGTTCGGCAGCGACCCTGAGGACGTTGATTATCAGCGTGGTTTCAACGGGGACATTACCGAGCGTAACATCTCGCTGTATGGTGCGCATGATAACTTCTTGAGCCAGCAAGCTCAGAAGGGCGCTATCATGAACAGCCGAGTGAAACTc                                                                <  1:220299/287‑1 (MQ=255)`  
                                                                                                                                                                                                                                                  `aCTCCGAGTCAAGACTGGTCGTAACGCTGCGTATCTTGTGGACGATGACGTTATGCAGCAGATAAAAGAGGGTGTCTTCCGTACTCGCGAAGAGATGGAAGAGTATCGCCATAGTCGCCTTCAAGAGGGCGCTAAGGTATACGCTGAGCAGTTCGGCATCGACCCTGAGGACGTTGATTATCAGCGTGGTTTCAACGGGGACATTACCGAGCGTAACATCTCGCTGTATGGTGCGCATGATAACTTCTTGAGCCAGCAAGCTCAGAAGGGCGCTATCATGAACAGCCGAGTGGAACTCAAc                                                             <  1:8588/301‑1 (MQ=255)`  
                                                                                                                                                                                                                                                        `aGTCAAGACTGGTCGTAACGCTGCGTATCTTGTGGACGATGACGTTATGCAGCAGATAAAAGAGGGTGTCTTCCGTACTCGCGAAGAGATGGAAGAGTATCGCCATAGTCGCCTTCAAGAGGGCGCTAAGGTATACGCTGAGCAGTTCGGCATCGACCCTGAGGCCGTTTATTATCAGCGTGGTTTCAACGGGGCCTTTACGCAGCTTAACATCTCCGTTTTTGTTCCGCTTGAAAACTTTTTTACCCCGCAAGCTCCAAAGGCCGATTACATAAACATCCGAGTGGAACTCAActtagcc                                                       >  1:304137/1‑295 (MQ=255)`  
                                                                                                                                                                                                                                                            `aaaaatGGTCGTAACGCTGCGTAACTTGTGGACGAGGAAGTTAGGCAGCAGATAAAAGAGGGTGTCTTCCGTACGCGCGAAGAGATGGAAGAGTATCGCCATAGTCGCATTCAAGAGGGGGATAAGGTGTACGCTGAGCAGTTCGGCATCGACCCTGAGGACGTTGATGATCAGCGTGGTTTCAACGGGTACATTACCGGGCGTAACATCTCGCTGTATGGTGCGCATGATAACTTCTTGATCCAGCAAGCTCAGAAGGGCGCTATCATGAACAGCCGAGTGGAACTCAACGGTGTCCTTc                                                   <  1:160636/296‑1 (MQ=255)`  
                                                                                                                                                                                                                                                                 `tGGTCGTAACGTTGCGTATCTTGTGGACGATGACGTTATGCAGCAGATAAAATAGGGTGTATTCCGTACTCGCGAAGGGATGGAAGGGTATCGCCATAGTCGCCTTCAAGAGGGCGCTAAGGTATACGCTGAGCAGTTCGGCATCGACCCTGAGGACGTTGATTATCAGCGTGGTTTCAACGGGGACATTACCGAGCGTAACATCTCGCTGTATGGTGCGCATGATAACTTCTTGAGCCAGCAAGCTCAGAAGGTCGCTATCGTGAACAGCCGAGTGGAACTCAACGGTGTCCTTAAAGAc                                              <  1:120235/301‑1 (MQ=255)`  
                                                                                                                                                                                                                                                                 `gggTCGTAACGCTGCGTATCTTGTGGACGATGACGTTATGCAGCAGATAAAAGAGGGTGTCTTCCGTACGCGCGAAGAGATGGAAGAGTATCGCCATAGTCGACTTCAAGAGGGCGCTAAGGTAGACGCTGAGCAGTTCGGCATCGACCCTGAGGACGTTGATTATCAGTGTGGTTTCAACGGGGACATTACCGAGCGTAACATCTCGCTGTATGGTGCGCATGATAACTTCTTGAGCCAGCAAGCTCAGAAGGGCGCTATCATGAACAGCCGAGTGGAACTCAACGGTGTCCTTAAAGAc                                              <  1:116263/300‑1 (MQ=255)`  
                                                                                                                                                                                                                                                                   `gTCGTAACGCTGGGTATCTTGTGGACGATGAGGTTATGGAGCAGATAAAAGAGGTTGTCTTCCGGACTCGCGAAGAGATGGAAGAGTATCGCCATAGTCGCCTTCAAGAGGGCGCTAAGGTATACGCTGAGCAGTTCGGCATCGACCGTGAGGACGTTGATTATAAGCGTGGTTTCAAGGGGGACAATACCGAGCGTAACATCTCGCTGTATGGTGCGCATGATAACTTCTTGAGCCAGCAAGCTCAGAAGGGCGGTATCATGAACAGCCGAGTTGAACTCAACGGTGTCCTTAAAGAccc                                            <  1:381049/301‑1 (MQ=255)`  
                                                                                                                                                                                                                                                                   `gTCGTAACGCTGCGTATCTTGTGGACGATGACGTTATGCAGCAGATAAAAGAGGGTGTCTTCCGTACTCGCGAAGAGATGGAAGAGTATCGCCATAGTCGCCTTCAAGAGGGCGCTAAGGTATACGCTGAGCAGCTCGGCATCGACCCTGAGGACGTTGATTATCAGCGTGGTTTCAACGGGGACATTACCGAGCGTAACATCTCGCTGTATGGTGCGCATGATAACTTCTTGAGCCAGCAAGCTCAGAAGGGCGCTATCATGAACAGCCGAATGGAACTGAACGGTGTCCTTCAAGAccc                                            >  1:344196/1‑301 (MQ=255)`  
                                                                                                                                                                                                                                                                         `ataagagacagcTTGTGGACGATGACGTTATGCAGCAGATAAAAGAGGGTGTCTTCCGTACTCGCGAAGAGATGGAAGAGTATCGCCATAGTCGCCTTCAAGAGGGCGCTAAGGTATACGCTGAGCAGTTCGGCATCGACCCTGAGGACGTTGATTATCAGCGTGGTTTCAACGGGGACATTACCGAGCGTAACATCTCGCTGTATGGTGCGCATGATAACTTCTTGAGCCAGCAAGCTCAGAAGGGCGCTATCATGAACAGCCGAGTGGAACTCAACGGTGTCCTTAAAGACCCTGatat                                      <  1:124494/290‑1 (MQ=255)`  
                                                                                                                                                                                                                                                                           `gctgctTATCTTGTGGACGATAACGTTATGAAGCAGATAAAAGAGGGTGTCTTCCGTAATCGCAAAGGGATGGAAGAGTATAGCCATAGTCGCCTTCAAGAGGGGGCTAAGGTATACGCTGAGCAGTTCGGCATCGACCCTGAGGACGTTGATTATCAGCGTGGTTTCAACTGTGACATTACCGAGCGTAACATCTCGCTGTATGGTGCGCATGATAACTTCTTGAGCCAGCAAGCTCATAAGGGCGCTATCATTAACAGCCGAGTGGAACTCAACGGTGTCCTTCAAGACCCTGATAtgc                                    <  1:499673/301‑1 (MQ=255)`  
                                                                                                                                                                                                                                                                           `gccaggTATCTTGTGGACGATGACGTTATGCAGCAGATAAAAGAGGGTGTCTTCCGTACTCGCGAAGAGATGGAAGAGTATCGCCATAGTCGCCTTCAAGAGGGCGCTAAGGTATACGCTGAGCAGTTCGGCATCGACCCTGAGGACGTTGATTATCAGCGTGGTTTCAACGGGGACATTACCGAGCGTAACATCTCGCTGTATGGTGCGCATGATAACTTCTTGAGCCAGCAAGCTCAGAAGGGCGCTATCATGAACAGCCGAGTGGAACTCAACGGTGTCCTTAAAGACCCTGATAtgc                                    <  1:495950/296‑1 (MQ=255)`  
                                                                                                                                                                                                                                                                            `cTGCGTATCTTGTGGACGATGACGTTATGCAGCAGATAAAAGAGGGTGTCTTCCGTACTCGAGAAGAGATGGAAGAGTATCGCCATAGTCGCCTTCAAGAGGGCGCTAAGGTATACGCTGAGCAGTTCGGCATCGACCCTGAGGACGTTGATTATCAGCGTGGTTTCAACGGGGACATTACCGAGCGTAACATCTCGCTGTATGGTGCGCATGATAACTTCTTGAGCCAGCAAGCTCAGAAGAGCGCTCTCATGAACAGCCGATGGGAACTCAACGGTGTCATTCAATACCCTGCTAtgct                                   >  1:357745/1‑301 (MQ=255)`  
                                                                                                                                                                                                                                                                              `gCGTATCTTGTGGACGATGACGTTATGCAGCAGATAAAAGAGGGTGTCTTCCGTACTCGCGAAGAGATGGAAGAGTATCGCCATAGTCGCCTTCAAGAGGGCGCTAAGGTATACGCTGAGCAGTTCGGCATCGCCCCTGAGGCCGTTGATTATCAGCGTGGTTTCAACGGGGACATTACCGAGCGTACCCTCTCGCTGTATGGTGCGCATGATAACTTCTTGAGCCAGCACGCCCAGAAGGGCGCTATCATTAACAGCCGAGTGGAACTCAACGGTGTCCTTCAAGACCCTGATAtgcctg                                 >  1:285311/1‑298 (MQ=255)`  
                                                                                                                                                                                                                                                                                `gTATCTTGTGGACGATGACGTTATGCAGCAGATAAAAGTGGGTGTCTTCCGTACTCGCGAAGAGATGGAAGAGTATCGCCATAGTCGCCTTCAAGAGGGCGCTAAGGTATACGCTGAGCAGTTCGGCATCGACCCTGAGGACGTTGATTATCAGCGTGGTTTCAACGGGGACATTACCGAGCGTAACATCTCGCTGTATGGTGCGTATGATAACTTCTTGAGCCAGCAAGCTCAGAAGGGCGCTATCATGAACAGCCGAGTGGAACTCAACGGTGTCCTTCAAGACCCTGATATGCTGcgt                               >  1:410847/1‑301 (MQ=255)`  
                                                                                                                                                                                                                                                                                `gTATCTTGTGGACGATGACGTTATGCAGCAGATAAAAGAGGGTGTCTTCCGTACTCGCGAAGAGATGGAAGAGTATCGCCATAGTCGCCTTCAAGAGGGCGCTAAGGTATACGCTGAGCAGTTCGGCATCGACACTGAGGACGTTGATTATCAGAGTGGTTGCAACGGGGACCTTACCGAGCGTAACATCTCGCTGTCTGGTGCGAATGATAACTTCTTTAGCCAGCAATCTCAGCATGGCGCTATCATGAACCGGCGAGTGGGACTCAACGGTGTCCTTCCAGCACCTGCATTGCTGcgt                               >  1:261173/1‑301 (MQ=255)`  
                                                                                                                                                                                                                                                                                       `gtgGATGATGACGTTATGCAGCAGATAAAAGAGGGTGTCTTCCGTACTCGCGAAGAGATGGAAGAGTATCGCCATAGTCGCCTTCAAGAGGGCGCTAAGGTATACGCTGAGCAGTTCGGCATCGACCCTGAGGACGCTGATTATCAGCGTGGTTTCAACGGGGACATTACCGAGCGTAACATCTCGCTGTATGGTGCGCATGATAACTTCTTGAGCCAGCAAGCTCAGAAGGGCGCTATCATGAACAGGCGAGGGGAACTCAACGGTGTCCTTCAAGACCCTGATATGCTGCGGC‑GTCCAg                       >  1:241486/1‑301 (MQ=255)`  
                                                                                                                                                                                                                                                                                           `aCGATGACGTTATGCAGCAGATAAAAGAGGGTGTCTGCCGTATTCGCGAAGAGATGGAAGAGTATCGCCATAGTCGCCTTCAAGAGGGAGCTAAGGGATACGCTGAGCAGTTCGGCATCGACCCTGAGGACGTTGATTATCTGCGTGGTTTCAACGGGGACATTACCGAGCGTAACATCTCGCTGTATGGTGCGCATGATAACTTCTTGAGCCAGCAAGCTCAGAAGGGCGCTATCATGAACAGCCGAGTGGAACTCAACGGTGTCCTTAAAGACCATGATATGCTGCGTC‑GTCCAGActc                   <  1:425629/301‑1 (MQ=255)`  
                                                                                                                                                                                                                                                                                            `cGATGACGTTATGCAGCAGATAAAAGAGGGTGTCTTCCGTACTCGCGAAGAGATGGAAGAGTATCGCCATAGTCGCCTTCAAGAGGGCGCTAAGGTATACGCTGAGCAGTTCGGCATCGACCCTGAGGACGTTGATTATCAGCGTGGTTTCAACGGGGACATTACCGAGCGTAACATCTCGCTGTATGGTGCGCATGATAACTTCTTGAGCCAGCAAGCTCAGAAGGGCGCTATCATGAACAGCCGAGTGGAACTCAACGGTGTCCTTCAAGACCCTGATATGCTGCGTC‑GTCCAGActct                  >  1:335436/1‑301 (MQ=255)`  
                                                                                                                                                                                                                                                                                              `aTGACGTTATGCAGCAGATAAAAGAGGGTGTCTTCCGTACTCGCGAAGAGATGGAAGAGTATCGCCATAGTCGCCTTCAAGAGGGCGCTAAGGTATACGCTGAGCAGTTCGGCATCGACCCTGAGGACGTTGATTATCAGCGTGGTTTCAACGGGGACATTACCGAGCGTAACATCTCGCTGTATGGTGCGCATGATAACTTCTTGAGCCAGCAAGCTCAGAAGGGCGCTATCATGAACAGCCGAGTGGAACTCAACGGTGTCCTTCAAGACCCTGATATGCTGCGTC‑GTCCAGActcctg                >  1:232425/1‑298 (MQ=255)`  
                                                                                                                                                                                                                                                                                                 `aCGTTATGCAGCAGATAAAAGAGGGTGTCTTCCGTACTCGCGAAGAGATGGAAGAGTATCGCCATAGTCGCCTTCAAGAGGGCGCTAAGGTATACGCTGAGCAGTTCGGCATCGACCCTGAGGACGTTGATTATCAGCGTGGTTTCAACGGGGACATTACCGAGCGTAACATCTCGCTGTATGGTGCGCATGATAACTTCTTGAGCCAGCAAGCTCAGAAGGGCGCTATCATGAACAGCCGAGTGGAACTCAACGGTGTCCTTCAAGACCCTGATATGCTGCGTC‑GTCCAGACTCTGCTGa             >  1:104901/1‑301 (MQ=255)`  
                                                                                                                                                                                                                                                                                                  `cGTTATGCAGCATATAAAAGAGGGTGTCTTCCGTACTCGCGAAGAGATGGAAGAGTATCGCCATAGTCGCCTTCAAGAGGGCGCTAAGGTATACGCTGAGCAGTTCAGCATCGACCCTGAGGACGTTGATTATCAGCGTGGATTCAAAGGGGACATTACCGAGCGTAACATCTCGCTGTATGGTGCGCATGATAACTTCTTGAGCTAGCAAGCTCAGAAGGGCGCTATCATGACCAGCAGAGTGCAACTCAACGGTGGCCTTTCAGACCCTGATAATCTTCGTCAGTCAAG‑CTCTGCTGAc            >  1:110284/1‑301 (MQ=255)`  

                                                                                                                                                                                                                                                                                                            `|`                                                                                                                                                                                                                                                                                                               
`GGAGGTATGGGCTATCGTGCAGCAACCACTCAGGCCGAACAGCCAAGGTCAAGCCTATTGGACACCATTGGTCGGTTCGCTAAGGCTGGTGCCGATATGTATACCGCTAAGGAACAACGAGCACGAGACCTAGCTGATGAACGCTCTAACGAGATTATCCGTAAGCTGACCCCTGAGCAACGTCGAGAAGCTCTCAACAACGGGACCCTTCTGTATCAGGATGACCCATACGCTATGGAAGCACTCCGAGTCAAGACTGGTCGTAACGCTGCGTATCTTGTGGACGATGACGTTATGCAGAAGATAAAAGAGGGTGTCTTCCGTACTCGCGAAGAGATGGAAGAGTATCGCCATAGTCGCCTTCAAGAGGGCGCTAAGGTATACGCTGAGCAGTTCGGCATCGACCCTGAGGACGTTGATTATCAGCGTGGTTTCAACGGGGACATTACCGAGCGTAACATCTCGCTGTATGGTGCGCATGATAACTTCTTGAGCCAGCAAGCTCAGAAGGGCGCTATCATGAACAGCCGAGTGGAACTCAACGGTGTCCTTCAAGACCCTGATATGCTGCGTC‑GTCCAGACTCTGCTGACTTCTTTGAGA  >  NC_001604.1/28391‑28991`

  

Alignment Legend

Aligned base mismatch/match (shaded by quality score): `ATCG/ATCG` `< 3 ≤` `ATCG/ATCG` `< 9 ≤` `ATCG/ATCG` `< 16 ≤` `ATCG/ATCG` `< 31 ≤` `ATCG/ATCG` `< 38 ≤` `ATCG/ATCG`

Unaligned base: `atcg`    Masked matching base: `atcg`    Alignment gap: `‑`    Deleted base: `‑`
